# Insulin glargine versus other types of basal insulin–clinical and tumor characteristics in patients with breast carcinoma

**DOI:** 10.1186/1756-0500-6-416

**Published:** 2013-10-16

**Authors:** Nikola Besic, Nika Satej

**Affiliations:** 1 Institute of Oncology Ljubljana, Zaloska 2, 1000 Ljubljana, Slovenia; 2 Community Health Centre Ljubljana, Krziceva 10, 1000 Ljubljana, Slovenia

**Keywords:** Diabetes mellitus, Insulin, Oncology, Breast

## Abstract

**Background:**

Recent epidemiological studies have suggested that some insulin analogues could be associated with an increased risk of cancer. The aim of this retrospective study was to examine whether patients with diabetes mellitus (DM) using insulin glargine have a higher tumor stage of breast carcinoma in comparison to patients using other types of insulin.

**Methods:**

We performed a chart review of 79 surgically treated breast carcinoma patients (mean age of 66.5 years; range 38-86 years) who were on insulin. Insulin glargine was used in 13 patients, while the other 66 patients were on other types of insulin. Clinical and histopathology characteristics of patients on glargine versus other types of insulin were compared using a chi-square test and non-parametric statistical analysis.

**Results:**

DM type 1 and DM type 2 was present in 14 and 65 patients, respectively. The mean tumor size was 2.98 cm. The TNM tumor stage at diagnosis was not higher among patients on glargine compared to patients on other types of insulin (T1/T2 85% vs. 68%, T3/T4 15% vs. 32%, p = 0.32; N1 54% vs. 58%, p = 0.80; M1 8% vs. 6%, respectively). No significant differences between both study groups (glargine vs. other types of insulin) were found in the ages of the patients, their BMI, tumor histology, grade, number of metastatic lymph nodes, hormone receptors or HER-2 status.

**Conclusion:**

We could not show that patients with DM using insulin glargine have a higher tumor stage of breast carcinoma in comparison to those using other types of insulin.

## Background

Epidemiological studies show that patients with diabetes mellitus (DM) have an increased risk of breast carcinoma [1]. It is known that anti-diabetic drugs may have an impact on breast carcinoma [2,3]. Patients with type 2 diabetes exposed to sulfonylurea or exogenous insulin had a significantly increased risk of cancer-related mortality compared with patients exposed to metformin [4]. Recent epidemiological studies have suggested that some insulin analogues could be associated with an increased risk of cancer [5]. Observational epidemiological data reported by Hemkens et al. [5] raised safety concerns about the mitogenic properties of insulin glargine in patients with diabetes. In a recent review of the literature, Smith and Gale stated that it is currently impossible to extrapolate from the in vitro to the in vivo situation with any confidence [6].

There are conflicting data about the impact of insulin glargine on breast cancer incidence [6-8]. Glargine, detemir and lispro, unlike regular insulin, exhibit in vitro proliferative and anti-apoptotic activities in a number of cancer cell lines [9]. In vitro studies on breast cancer cell lines showed that the serum of patients with diabetes was a slightly stronger mitogenic when using glargine as compared to detemir or insulin with intermediate duration for action [10]. But data is scarce in the literature about clinical and histopathological characteristics of tumors in patients with breast carcinoma and DM. Our hypothesis was that the use of glargine has an impact on the higher mitogenic effect on tumor cells and consecutively on a faster progression of breast carcinoma in comparison to other types of insulin. The aim of this retrospective study was to examine whether patients with DM using insulin glargine have a higher tumor stage of breast carcinoma in comparison to patients using other insulin.

## Subjects and Methods

Altogether 252 patients with DM were surgically treated because of invasive breast carcinoma at a single institution from 2005-2011. A chart review of these 79 breast carcinoma female patients (mean age of 66.6 years; range 38-86 years) who were on insulin was performed. Insulin glargine was used in 13 patients, while the other 66 patients were on other types of insulin.

The data on clinical and histopathology characteristics: the patients’ age, body mass index (BMI), TNM tumor stage, number of metastatic lymph nodes, presence of estrogen and progesterone receptors and HER-2 expression were collected. The tumor stage, presence of regional metastases, distant metastases and residual tumor after surgery were assessed by the TNM clinical classification system according to UICC criteria [11] from 2007. BMI was calculated as weight/height^2^ (kg/m^2^). Co-morbidity was evaluated by the American Society of Anaestiologists (ASA score) [12].

In this study, routine final pathology reports were used. Histological slides were examined by six pathologists, experienced in breast pathology. Sentinel lymph nodes were examined by frozen section, immunohistochemistry and paraffin section. If the sentinel nodes turned out to be tumor-free, no further axillary surgery was recommended. If sentinel lymph nodes showed metastases in the frozen section, the patient underwent axillary dissection during the same surgical procedure. In cases of malignant involvement only in paraffin section or immunohistochemistry re-operation for axillary dissection was performed. For the purposes of this study estrogen-receptors and progesterone-receptors were considered positive if 10% or more tumor cells had a positive stain. The status of HER-2 receptors was determined by imunohistocemistry and the FISH method. Both methods had to show a positive result in order to classify the tumor as HER-2 positive.

Factors recorded for this study included surgical breast cancer treatment (breast conserving operation vs. mastectomy), axillary surgery (sentinel lymph node biopsy vs. axillary dissection), adjuvant chemotherapy, hormonal treatment and/or treatment with Trastuzumab.

The study has been reviewed by the appropriate ethics committee and has been performed in accordance with the ethical standards laid down in an appropriate version of the 1964 Declaration of Helsinki. The Institutional Review Board and Ethics Committee of the Institute of Oncology Ljubljana have approved the study. Our study was conducted with the understanding and consent of the human subjects.

### Statistical methods

The characteristics of patients and their treatments were compared using contingence tables. Because of the small number of patient groups, the ages of the patients, their BMI, tumor size and number of metastatic lymph nodes were compared using a Mann–Whitney rank-sum test. For statistical analysis SPSS 16.0 for Windows was used.

In order to estimate the probability (power) to reject the null hypothesis a “PS: Power and Sample Size Calculations” Software (Version 3.0, January 2009) was used. An uncorrected chi-squared statistic to evaluate the null hypothesis was used. The Type I error probability associated with this test of this null hypothesis was 0.05. In our study there were 13 experimental subjects and 66 control subjects. The true failure rate for experimental subjects was 0.15, while the failure rate among our controls was 0.32. We are able to reject the null hypothesis that the failure rates for experimental and control subjects are equal with probability (power) 0.190. Altogether 65 experimental subjects and 305 control subjects are needed in order to be able to reject the null hypothesis with probability (power) 0.8.

## Results

The mean age of the patients, their BMI, tumor size and number of metastatic lymph nodes was 66.5 years, 29.9 kg/cm^2^, 2.98 cm and 2.65, respectively. The characteristics of patients treated with glargine and other types of insulin are presented in Table 1. Tumor-specific therapy and the outcome of both groups of patients are presented in Table 2.

**Table 1:** Tumor and demographic charcateristics of 79 patients with diabetes treated with glargine and other types of insulin

**Factor**	**Subgroup**	**Patients on glargine**	**Patients on other types of insulin**	**p-value**
		**(N = 13)**	**(N = 66)**	
**Median age (years)**		63	67	0.30
**Median tumor size (mm)**		22	20	0.93
**Median BMI (kg/m**^**2**^**)**		28.6	29.75	0.51
**Median ASA score**		2	2	0.95
**Median number of metastatic lymph nodes**		1	1	0.58
**Mitoses (Median)**		2	2	0.72
**Age (years)**	70 or less	10	42	0.52
	71 or more	3	24	
**BMI (kg/m**^**2**^**)**	less than 30	2	12	0.74
**(N = 76)**	30 or more	10	52	
**American Society of Anesthesiologists score**	1	0	1	0.89
	2	7	34	
**(N = 74)**	3	5	27	
**Contralateral breast carcinoma**	No	13	64	1.00
	Yes	0	2	
**Another malignancy**	No	12	58	1.00
	Yes	1	8	
**Type of diabetes mellitus**	DM type 1	4	10	0.23
	DM type 2	9	56	
**Therapy with metformine**	No	11	54	1.00
	Yes	2	12	
**Therapy with sulfonylurea**	No	12	58	1.00
	Yes	1	8	
**pT tumor stage**	pT1	5	31	0.22
	pT2	6	14	
	pT3	1	4	
	pT4	1	17	
**T3 or T4 stage**	pT1 or pT2	11	45	0.32
	pT3 or pT4	2	21	
**N stage**	pN0	6	28	0.80
	pN1 or pN2	7	38	
**Number of metastatic lymph nodes**	0	6	28	0.95
	1–3	4	20	
	4 or more	3	18	
**M stage**	M0	12	62	1.00
	M1	1	4	
**Type of invasive carcinoma**	Ductal	10	57	0.41
	Lobular or other types	3	9	
**Tumor differentiation**	Well or moderate	6	35	0.65
	Poor	7	31	
**Estrogen hormone status (10% or more)**	Positive	10	61	0.12
	Negative	3	5	
**Progesteron hormone status (10% or more)**	Positive	8	49	0.35
	Negative	5	17	
**HER-2**	Negative	13	62	1.00
	Positive	0	4	

**Table 2:** Carcinoma-related treatment of patients with diabetes in group of patients on glargine and other types of insulin

**Factor**	**Subgroup**	**Patients on glargine**	**Patients on other types of insulin**	**p-value**
		**(N = 13)**	**(N = 66)**	
**Breast surgical procedure**	Quadrantectomy or lumpectomy Mastectomy	7	23	0.20
		6	43	
**Axillary surgical procedure**	Sentinel node biopsy	5	24	0.78
	Lymphadenectomy	8	42	
**Adjuvant Chemotherapy**	No	9	50	0.72
	Yes	4	16	
**Adjuvant Hormone therapy**	No	3	6	0.17
	Yes	10	60	
**Adjuvant Trastuzumab**	No	13	62	1.00
	Yes	0	4	
**Adjuvant Radiotherapy**	No	5	32	0.49
	Yes	8	34	
**Outcome**	Alive	13	63	1.00
	Died of disease	0	3	

The TNM stage at diagnosis was not higher among patients on glargine, compared to patients on other types of insulin (p = 0.32). No significant differences between the study groups were found in the ages of the patients, their BMI, tumor histology, grade, number of metastatic lymph nodes, hormone receptor status or HER-2 status (Table 1).

## Discussion

Epidemiological data show that the patients with DM have a worse prognosis and a higher risk of cancer-related mortality in comparison to patients without DM [13,14]. In accordance with this finding is the study of Unterburger et al. [15] who reported a correlation between the presence of diabetes and metastatic disease. Similarly Wolf et al. [16] found that at presentation patients with breast cancer and DM had a more advanced stage in comparison to patients without DM. They investigated the effects of DM type 2 on breast cancer at presentation in 79 consecutive patients with DM and breast cancer by comparison with 158 age-matched patients who did not have DM [16]. This finding could not be attributed to parity, family history of breast cancer, obesity, or other risk factors for breast cancer [17]. But Guastamacchia et al. [18], in post-menopausal patients, found no association between DM and breast cancer stage or hormone-receptor status comparing 77 patients with DM and 578 controls. Furthermore, they found that tumors of insulin-treated women had a lower proliferative activity of breast carcinoma than non-insulin treated ones [18].

In vitro studies on breast cancer cell lines showed that the serum of patients with diabetes was a stronger mitogenic when using glargine as compared to other types of insulin [10]. Our hypothesis was that the use of glargine has an impact on the mitogenic effect on tumor cells and so on the faster progression of breast carcinoma in comparison to other types of insulin. Therefore, the aim of our study was to examine whether patients with DM using insulin glargine have a higher tumor stage of breast carcinoma in comparison to patients using other types of insulin. We could not show that patients with DM using insulin glargine have a higher tumor stage of breast carcinoma in comparison to those using other types of insulin. Furthermore, no significant differences between the study groups were found in the ages of the patients, their BMI, tumor histology, number of metastatic lymph nodes, hormone receptor status or HER-2 status. Also factors which correlate with the mitogenic effect did not differ in glargine users in comparison to patients on other types of insulin. Neither the mitosis rate of tumors or tumor grade was significantly different in both groups of patients. Based on these results we believe that insulin glargine does not express a mitogenic effect in vivo in breast cancer patients.

Of course there are several limitations of our study. It is retrospective, observational and nonrandomized. Because of the small number of patients in our study we were able to reject the null hypothesis that the failure rates for experimental and control subjects are equal with probability (power) of only 0.190. Besides, because of the small number of patients we could not take into account the dose of insulin, in spite of the fact that we were aware that the possibility of association between cancer and higher glargine doses suggests that dosages should always be considered when assessing the possible association of insulin and its analogues with cancer [19]. Furthermore, different combinations of anti-diabetic drugs and types of insulin and doses of glargine and other types of insulin were used in our patients. Moreover, our glargine group comprised only thirteen patients. Yet, to our knowledge, it is the largest group of patients on glargine compared to other types of insulin in which the TNM stage and histological characteristic of tumors were compared. All the patients with breast carcinoma and DM on any type of insulin were included in our study in order to avoid selection bias.They were treated in a seven year period at a single tertiary cancer comprehensive center, in which about 800 surgical procedures because of breast carcinoma are performed annually. About two thirds of all breast carcinoma patients from our country are surgically treated at our center, therefore we believe that our study adequately represents the Slovenian breast cancer population.

Interestingly, despite both DM and breast carcinoma being common diseases, the data about histopathology characteristics and the extent of the disease in these patients in the literature are scarce and conflicting. In comparison to data reported by Jiralensong et al. [3], our patients are older and have a lower mean BMI index. Furthermore, our patients have a lower proportion of triple negative tumors and HER-2 positive tumors, probably because the majority of our patients are postmenopausal. On the other hand, the characteristics of our patients are comparable to patients reported by Wolf et al. [16]. The mean age of patients, their BMI index, rate of estrogen positive tumors, progesterone positive tumors and HER-2 positive tumors is 66.5 years, 64.9 years, 29.9 kg/cm^2^ and 29.7 kg/cm^2^, 0.89, 0.75, 0.72, 0.44, 0.05 and 0.11 in the patients from our and their study, respectively. Unfortunately, Wolf et al. [16] did not analyze the impact of anti-diabetic treatment on the cancer tumor stage of patients with DM.

The direct biological effects of DM for patients with breast cancer are difficult to define, mainly because of the presence of confounding factors such as obesity, old age, co-morbidity, and differences in screening use or treatment allocation [17]. We agree with reports from the literature that anti-diabetic drugs may have an impact on breast carcinoma [2,3]. But, based on the results of our study, we could not confirm that insulin glargine in comparison to other types of insulin causes faster tumor progression. We share the opinion of Hemkens et al. [5] that prospective long-term studies are needed to further evaluate the safety of insulin analogues.

## Conclusion

The TNM tumor stage of patients with breast carcinoma and diabetes mellitus at diagnosis was not higher among 13 patients on glargine compared to 69 patients on other types of insulin. Furthermore, there was no significant difference in the mitosis rate of tumors or tumor grade among both groups of patients.

## Competing interests

Authors declare that there is no conflict of interest that could be perceived as prejudicing the impartiality of this paper.

## Authors’ contributions

NB participated in the design of the study, partially collected data and performed the statistical analysis. NS participated in collecting data and drafted the manuscript. Both authors read and approved the final manuscript.
